# Parameters of Starch Granule Genesis in Chloroplasts of *Arabidopsis thaliana*

**DOI:** 10.3389/fpls.2018.00761

**Published:** 2018-06-05

**Authors:** Irina Malinova, Hadeel M. Qasim, Henrike Brust, Joerg Fettke

**Affiliations:** Biopolymer Analytics, University of Potsdam, Potsdam, Germany

**Keywords:** starch biosynthesis, starch granule biogenesis, starch synthase, plastidial phosphorylase, malto-oligosaccharides

## Abstract

Starch is the primary storage carbohydrate in most photosynthetic organisms and allows the accumulation of carbon and energy in form of an insoluble and semi-crystalline particle. In the last decades large progress, especially in the model plant *Arabidopsis thaliana*, was made in understanding the structure and metabolism of starch and its conjunction. The process underlying the initiation of starch granules remains obscure, although this is a fundamental process and seems to be strongly regulated, as in Arabidopsis leaves the starch granule number per chloroplast is fixed with 5-7. Several single, double, and triple mutants were reported in the last years that showed massively alterations in the starch granule number per chloroplast and allowed further insights in this important process. This mini review provides an overview of the current knowledge of processes involved in the initiation and formation of starch granules. We discuss the central role of starch synthase 4 and further proteins for starch genesis and affecting metabolic factors.

## Introduction

Starch is one of the most abundant biopolymers on earth. The entire process of starch biosynthesis encompasses: the generation of a suitable primer (glucosyl acceptor); the biosynthesis of a glucosyl donor (in most cases ADPglucose); the elongation of the primer by repetitive glucosyl transfer reactions via starch synthases (SS) and most probably via plastidial phosphorylase (see below); the introduction of branching points via branching enzymes and the removal of excess branching points via debranching enzymes; and the ordering of the glucans to the final physical organization. Starch granule synthesis in green algae, mosses, ferns, and higher plants occurs inside the plastids. In many other eukaryotic algae starch is formed in the cytosol, which is assumed to represent an earlier evolutionary state (Colleoni et al., 2010; Ball et al., 2011). This review will focus on the plastidial starch biosynthesis, more precisely the induction of the starch biosynthesis in *Arabidopsis thaliana* leaves.

To some extent, the initial steps of the starch biosynthesis, the generation of a suitable primer (a glucosyl acceptor, most likely a glucan), and the further elongation of the glucosyl acceptor by the action of glucosyl transferases is not precise to separate. The first elongation processes as well as the first branching/debranching events does not immediately result in a native starch granule rather generate a polyglucan. Furthermore, SS specifically interact with branching enzymes during the process of starch synthesis (Brust et al., 2014). But only following multiplicity of these reactions a starch granule is formed. Therefore, in this review we divide the starch synthesis in two steps. We define **starch initiation** in a strict sense as a process in which a primer-like structure/initiation complex is generated, that can in principle be further used for polymerization reactions. This also includes the possibility that no starch granules are detectable since the primer-like structure/initiation complex is not further synthesized. The term **starch formation** indicate when these potential primer-like structures/initiation complexes are used to form starch granules, and so starch granules are detectable in chloroplasts.

## Important Role of Starch Synthase 4

Starch synthases (SSs) are the enzymes responsible for elongation of amylose and amylopectin chains. These enzymes transfer glucosyl residue from ADPglucose to the non-reducing end of a preexisting glucan. It was discussed whether SSs are able to synthesize glucan chains without a glucan primer (Szydlowski et al., 2009; D’Hulst and Mérida, 2012). However, using heterologously expressed and purified SSs from Arabidopsis a primer was always required, that contained at least two glucosyl residues. Thus, maltose but not glucose was elongated by the SSs (Brust et al., 2013).

Analyses of SS mutants revealed that SSs have overlapping and in parts distinct functions during starch synthesis (Delvallé et al., 2005; Zhang et al., 2005, 2008; Roldán et al., 2007; Szydlowski et al., 2009, 2011). SS4 seems to be most important for formation of starch granules. Thus, the *ss4* mutant reveals mostly one starch granule per chloroplast (Roldán et al., 2007). However, other SS isoforms may partially substitute the SS4 function and so additional loss of SS3 further reduces the starch granule number in chloroplasts (Szydlowski et al., 2009; D’Hulst and Mérida, 2012). Interestingly, the number of starch granules in *ss4* is still flexible as the number shows variability in regards to growth conditions/developmental stage and enzymatic setup (see below). Thus, immature leaves of *ss4* have no detectable starch granules in contrast to mature leaves (Crumpton-Taylor et al., 2013). The number of starch granules per chloroplast depends of the length of the light phase as under continuous light the starch granule number increases to up to three (Malinova et al., 2017). Furthermore, the additional lack of α-amylase 3 (Seung et al., 2016) and an increased capacity for glucan synthesis via expression of a bacterial glycogen synthase (Crumpton-Taylor et al., 2013) result in a higher number of starch granules per chloroplast. An over expression of SS4 in Arabidopsis causes an elevated in an increased starch content, although an increase in starch granule number per chloroplast was not reported (Gamez-Arjona et al., 2011).

x

All five SSs in Arabidopsis share a core sequence of approximately 60 kDa at the C-terminus responsible for the catalytic activity. The N-terminal sequences are largely different. Interestingly, SS3 and SS4 possess a large N-terminal extension. The N-terminal region of SS4 is predicted to contain two long coiled-coil domains (Leterrier et al., 2008; D’Hulst and Mérida, 2012) and was reported to enable the association with the thylakoid membrane and interaction with thylakoid- and plastoglobule-bound fibrillin proteins (Gamez-Arjona et al., 2014; Raynaud et al., 2016). Furthermore, a subchloroplastidial localization of SS4 peripheral to starch granules was shown (Gamez-Arjona et al., 2014). Expression of the N-terminal region of SS4 in *ss4* did not result in alterations of the starch granule number. However, the expression of the C-terminal catalytically active region of SS4 in *ss4* resulted in an increased number of starch granules per chloroplast (Lu et al., 2018). It was shown that SS4 forms dimers necessary for its enzymatic activity and dimerization is mediated by a region located between the coiled-coil domain and the glycosyltransferase domain (Raynaud et al., 2016).

Summarizing, SS4 has impact on formation of starch granules and seems to be important for the generation of the regular number of granules per chloroplast. However, the observed reduced number of starch granules by the lack of SS4 can be in parts reversed by metabolic factors (see below).

## Additional Proteins Influencing the Starch Granule Number

Recently, a double mutant lacking the plastidial phosphorylase (PHS1; Zeeman et al., 2004) and the disproportionating enzyme 2 (DPE2; Chia et al., 2004; Lu and Sharkey, 2004; Fettke et al., 2006) was published (Malinova et al., 2014). Both parental lines have 4-7 starch granules per chloroplast similar to wild type. Interestingly, *dpe2*/*phs1* revealed mostly one starch granule per chloroplast when grown under 12 h light and 12 h dark conditions, similar to *ss4*. However, expression of SS4 was unaltered in this mutant (Malinova et al., 2014). The triple mutant *dpe2/phs1/ss4* also has only one starch granule per chloroplast when grown under 12 h light and 12 h dark (Malinova et al., 2017). More interestingly, when the light/dark regime was altered *ss4*, *dpe2/phs1*, and the triple mutant plants revealed variability in the starch granule number per chloroplast. An elongated light phase and continuous light resulted in an increased number of starch granules per chloroplast in all mutants but with different quantities. In *ss4* the number increased up to three granules whereas *dpe2/phs1* showed with 5-7 the wild type number of starch granules per chloroplast. The *dpe2/phs1/ss4* revealed an intermediate number of starch granules with 1-4 granules per chloroplast (Malinova et al., 2017). The observed increased number of starch granules in *dpe2/phs1* similar to wild type was supposed to be linked to the missing dark phase and the lack of starch degradation (Malinova et al., 2014). To test whether ongoing starch degradation in *dpe2/phs1* is necessary for the observed reduction of the starch granule number a triple mutant lacking the glucan, water dikinase (GWD; *sex1-8*; Ritte et al., 2002; Mahlow et al., 2014) in the background of *dpe2/phs1* was generated. GWD is involved in the phosphorylation/dephosphorylation cycle at the starch granule surface and is the key enzyme of the initiation of starch degradation (Hejazi et al., 2012; Mahlow et al., 2016). However, s*ex1-8* did not reveal a reduced starch granule number per chloroplast (Mahlow et al., 2014). Similarly, *dpe2/phs1/sex1-8* revealed 5-7 starch granules even when plants were grown at 12 h light and 12 h dark conditions (Malinova and Fettke, 2017). Thus, it was concluded that ongoing starch degradation is essential for the observed reduced number of starch granules in *dpe2/phs1* (Malinova and Fettke, 2017).

Recently, three new starch granule bound proteins were discovered sharing high homology and building up a new protein family, PROTEIN TARGETING TO STARCH (PTST; Seung et al., 2015, 2017). All three proteins contain conserved coiled coil domains and a CBM48. While PTST1 was shown to play an essential role in amylose synthesis by localizing granule bound SSs to starch granules (Seung et al., 2015), PTST2 and PTST3 were demonstrated to be involved in alteration of the number of starch granules per chloroplast (Seung et al., 2017). In *ptst2* a reduced starch granule number was observed with mostly one starch granule per chloroplast. PTST3 has partially redundant function to PTST2 but reduction of granule number was less pronounced in *ptst3* compared with *ptst2*. Furthermore, it was shown that an overexpression of PTST2 in wild type background resulted in an increased number of small granules. In contrast, an overexpression of PTST2 in *ss4* background resulted in a further reduction of the starch granule number per chloroplast. Interestingly, PTST2 was shown to interact with SS4 and longer soluble maltodextrins (Seung et al., 2017).

## Metabolic Implications of Variability in Starch Granule Number Per Chloroplast

For *ss4* as well as for *ptst2* and *ptst2/ptst3* an accumulation of ADPglucose was reported (Crumpton-Taylor et al., 2013; Ragel et al., 2013; Seung et al., 2017). The highest ADPglucose accumulation was observed for *ss4* (with ∼100-fold increase compared to wild type). *ptst2/ptst3* revealed also a high accumulation of ADPglucose (with ∼30-fold compared to wild type) that was twice of that of *ptst2* (Seung et al., 2017). This accumulation of ADPglucose, the substrate of SSs, is thought to show that the SS4 acts upstream of all other SSs and that the other SSs have limited activity when SS4 is lacking. However, *ss4/ss3* showed a similar high accumulation of ADPglucose as *ss4* (with ∼100-fold increase compared to wild type). *dpe2/phs1* and especially *dpe2/phs1/ss4* also revealed mostly one starch granule per chloroplast but showed only a very moderate increase in ADPglucose content (two- and fourfold, respectively, compared to wild type). Furthermore, *dpe2/ss4* and *phs1/ss4*, each having one starch granule per chloroplast, revealed totally different ADPglucose values (*dpe2/ss4*: approximately twofold, *phs1/ss4*: 250-fold increase compared to wild type). Under continuous light, an increase in the granule number per chloroplast was observed for *ss4*, *dpe2/phs1*, and *dpe2/phs1/ss4*. But these observations were not accompanied by a reduction in the ADPglucose amounts (Malinova et al., 2017). Thus, a simple direct connection of a high ADPglucose content and the lack of SS4 or a reduced granule number is not given.

Another factor influencing the starch granule number is probably the availability of soluble maltodextrins as glucan primers for the initiation of a starch granule. The generation and elongation of soluble maltodextrins is proposed to be important during granule initiation (Nakamura, 2015). It was postulated that longer soluble maltodextrins (e.g., maltodecaose), long enough to produce a helical secondary structure, are bound by PTST2. PTST2 further interacts with SS4 and initiate the synthesis of starch granules (Seung et al., 2017). Interestingly, increased levels of soluble glucans were detected in *ss4* (Crumpton-Taylor et al., 2013; Malinova et al., 2017). Most of these glucans were rather shorter with a degree of polymerization (DP) less than six. In *dpe2/phs1* and *dpe2/phs1/ss4* a massive increase in these short glucans was observed (Malinova et al., 2014, 2017). The origin of the chloroplastidial maltodextrins necessary for initiation of starch granules is unknown so far. However, the chloroplastidial maltodextrin pool is certainly also influenced by starch degradation processes.

**FIGURE 1:**
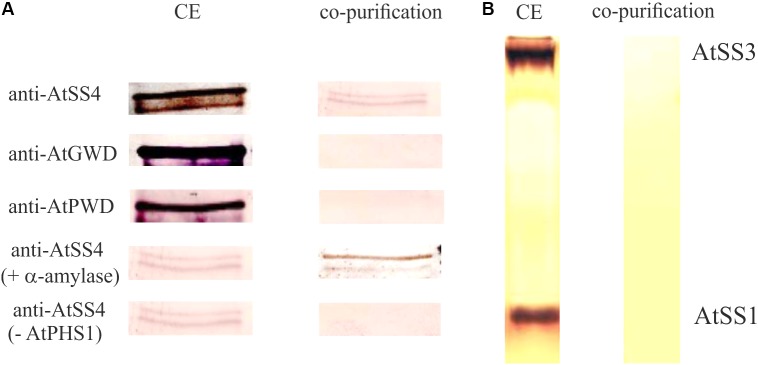
Interaction of starch synthase 4 and plastidial phosphorylase. **(A)** Purified C-terminal 6× His-taged recombinant plastidial phosphorylase protein from *Arabidopsis thaliana* (40 μg) expressed in *E. coli* was incubated with 200 μg protein crude extract (CE) isolated from Col-0 for 5 min at 21°C. Following purification of the recombinant plastidial phosphorylase using Ni-NTA agarose the plastidial phosphorylase containing fraction was analyzed by SDS-PAGE and the proteins were transferred to a membrane. The membrane was analyzed by antibodies specific for AtSS4, AtGWD, and AtPWD. In addition the recombinant plastidial phosphorylase and the protein crude extract were pre incubated with 20 units a-amylase for 10 min at 37° C prior incubation and purification, to exclude a potential interaction of AtPHS1 and AtSS4 via a bound glucan (AtSS4 + a-amylase). As control the experiment was performed identical but the recombinant plastidial phosphorylase was omitted (AtSS4-AtPHS1). **(B)** Following the purification as described in **(A)** the plastidial phosphorylase containing fraction was analyzed by native gel and activity staining for SSs (see also Brust et al., 2014). As control 50μg crude extract protein isolated from Col-0 was loaded on the native gel. Thus, no co-purification of plastidial phosphorylase with AtSS1 or AtSS3 was observed.

PHS1 could be involved in metabolism of the chloroplastidial maltodextrins and therefore in starch synthesis. Several indications for a principle involvement of PHS1 in starch synthesis were published. For *Chlamydomonas reinhardtii*, potato tubers, and for rice endosperm it was shown that the plastidial phosphorylase is involved in starch synthesis (Dauvillée et al., 2006; Orawetz et al., 2016) and in granule initiation (Satoh et al., 2008). Interestingly, the alteration of starch quantities and qualities in rice and potato mutants were exclusively detected when plants were grown under low temperature conditions. It was speculated that at low temperatures SSs cannot sufficiently substitute the lack of the plastidial phosphorylase (Fettke et al., 2012b; Orawetz et al., 2016). Furthermore, for endosperm starch evidences exist for a protein–protein interaction of the plastidial phosphorylase with branching enzyme and disproportionating enzyme 1 (DPE1) and production of longer soluble maltodextrins (Tetlow et al., 2004; Hwang et al., 2016; Nakamura et al., 2017). Interestingly, in Arabidopsis PHS1 interacts with SS4 (**Figure 1**; Qasim, 2018).

The reduced starch granule number per chloroplast observed in *dpe2/phs1* is not detected in the *phs1.* The single mutant revealed a starch granule number similar to wild type (Malinova et al., 2014, 2017). It could be speculated that other proteins compensate the lack of PHS1 but if in addition the starch turn-over is altered an effect on starch initiation could be observed. A balance between generation and degradation of maltodextrins could be important. In this regards, it can be supposed, that an additional lack of degradation enzymes such as α-amylase 3 (AMY3; Seung et al., 2016) and DPE2 (Malinova et al., 2014, 2017) influence this balance. An increase of the starch granule number per chloroplasts was detected in the *ss4/amy3* mutant, which point to a degradation of glucans in *ss4* and this degradation generates primers that can be used by other SSs. In this context it can also be seen that an introduction of the glycogen synthase from *Agrobacterium tumefaciens* (able to initiate and elongate glucan chains using ADPglucose as substrate; Ugalde et al., 2003) in *ss4* resulted in an increase in starch granule number per chloroplast by generating more of such primers (Crumpton-Taylor et al., 2013; Lu et al., 2018). In agreement with the observed binding of longer soluble maltodextrins by PTST2, it could be speculated that soluble maltodextrins longer than DP10 are involved in the starch granule initiation, as in *dpe2/phs1* no limitation for maltodextrins shorter than DP10 were observed (Malinova et al., 2014). Similarly, when in the background of *dpe2/phs1* the starch degradation was blocked by the additional lack of GWD, this glucan balance was again influenced and resulted in an increased number of starch granules with 5-7 granules per chloroplast similar to wild type (Malinova and Fettke, 2017). However, the additional lack of GWD in *ss4* has no effect on the starch granule number (Crumpton-Taylor et al., 2013). Interestingly, *phs1/mex1* double mutant lacking PHS1 and in addition the maltose exporter 1 [essential for export of the starch degradation product maltose (Niittylä et al., 2004) and thus it acts upstream to DPE2 (Fettke et al., 2012a)] in contrast to *dpe2/phs1* has a similar to wild type starch granule number per chloroplast. In regards to the soluble maltodextrins both mutants are different too, thus only a small increase in soluble maltodextrins (length up to DP10) was observed in *mex1/phs1* compared to wild type (Malinova et al., 2014). A further rather indirect indication of this balanced maltodextrin system is related to the observed dependency of the length of the light and dark phase on the starch granule number in *ss4*, *dpe2/phs1*, and *dpe2/phs1/ss4* (Malinova et al., 2017).

**FIGURE 2:**
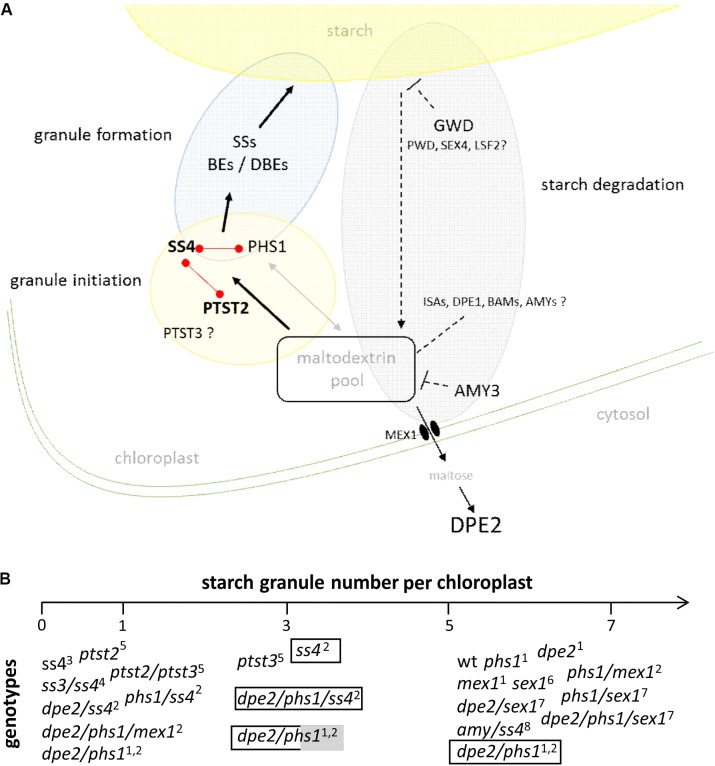
**(A)** Schematic presentation of starch granule synthesis. For starch granule initiation maltodextrins from the chloroplastidial maltodextrin pool are used by protein targeting to starch 2 (PTST2) and starch synthase 4 (SS4) to produce a granule initiation complex. A lack of the related enzymes massively influences the starch granule number. Enzymes related to initiation reveal protein-protein interaction as depicted by red links. The granule initiation complex is processed by SSs, branching (BEs), and debranching enzymes (DBEs) and a starch granule is formed. The chloroplastidial maltodextrin pool is affected by enzymes acting on glucans, efflux of carbohydrates to cytosol, and influx of carbohydrates via starch degradation. Thus, evidences were published for the involvement of glucan, water dikinase (GWD), disproportionating enzyme 2 (DPE2), α-amylase3 (AMY3), and plastidial phosphorylase (PHS1) during starch granule synthesis. For other enzymes acting on glucans (labeled with ?) the impact on the starch granule synthesis is lacking so far. ISAs- isoamylases; DPE1 and DPE2– disproportionating enzyme 1 and 2; BAMs – β-amylases; AMYs – α-amylases; PWD- phosphoglucan, water dikinase, SEX4 – starch excess 4, LSF2 – like starch excess4, MEX1 – maltose exporter 1 (see also Fettke et al., 2012a). Dashed arrow represents multiple enzymatic reactions. **(B)** Starch granule number per chloroplast in different genotypes. Plants were grown under 12 h ligth/12 h dark unless otherwise specified: transparent bar – continuous light, transparent/grey bar – 14 h light, 10 h dark.^1^Malinova et al., 2014; ^2^Malinova et al., 2017; ^3^Roldán et al., 2007; ^4^D’Hulst and Mérida, 2012; ^5^Seung et al., 2017; ^6^Mahlow et al., 2014; ^7^Malinova and Fettke, 2017; ^8^Seung et al., 2016.

Summarizing, as depicted in **Figure 2**, in a strict sense, the initiation of starch granules seems to be linked to maltodextrins serving as glucosyl acceptors. Increasing evidences reveal that these maltodextrin primers are influenced by the overall glucan metabolism in chloroplast. Thus, proteins such as cytosolic DPE2, PHS1, PTST2 and 3 are directly and/or indirectly involved in this process and so in starch granule initiation. Furthermore, this would also imply that the observed alteration of the starch granule number in *dpe2/phs1* as well as in *ptst2* and *ptst3* are upstream of the action of SS4. In agreement to this *dpe2/phs1* and *dpe2/phs1/ss4* contained mostly one starch granule. The double mutant revealed higher variability in the starch granule number in connection with metabolic alterations than the triple mutant. Thus, generation of the glucan primer seems more variable than formation of the starch granules regulated via SS4. Unfortunately, for both *ptst2* and *ptst3* such metabolic data are currently missing. However, the increase of the starch granule number when PTST2 is overexpressed in wild type but not when overexpressed in the *ss4* mutant is also in agreement with this assumption. Likewise, the observed increase of the starch granule number per chloroplast in *ss4* by expression of the glycogen synthase is conforming.

## Impact of a Reduced Starch Granule Number on Granule Morphology

Starch isolated from mutants with reduced number of starch granules per chloroplast reveal distinct alterations in the starch granule morphology. Starch granules from Arabidopsis leaves are thin and discoid. In contrast, starch from mutants lacking SS4 or DPE2/PHS1 show big and spherical starch granules (Crumpton-Taylor et al., 2013; Malinova et al., 2014, 2017).

It was reported that the N-terminal domain of SS4 influences the morphology of starch granules (Lu et al., 2018). Furthermore, the lack of PTST2 and PTST3 revealed single and spherical starch granules (Seung et al., 2017). Very interesting in this regards is again the *dpe2/phs1* mutant. When one starch granule per chloroplast was observed (under 12 h light/ 12 h dark regime) the granule was large and spherical, whereas when 5-7 starch granules were monitored (under continuous light) the starch granule morphology was similar to wild type (Malinova et al., 2017). It seems that the generation of a reduced number of starch granules number per chloroplasts is connected with formation of spherical rather than thin and flattened starch granules. However, also other starch-related mutants reveal alterations in morphology albeit these are less pronounced (SEX4; Kötting et al., 2009; LSF1; Comparot-Moss et al., 2010; GWD; Mahlow et al., 2014).

## Conclusion

In the last years we got more insights into starch granule biosynthesis. The appearance of starch granules is a rather complex mechanism influenced by various factors. According to this, it is very important to distinguish between the possibility to initiate starch and the formation of starch granules. It is quite clear that SSs, or more precisely SS4, is important in the formation of the typical number of starch granules per chloroplast in Arabidopsis (5-7, Crumpton-Taylor et al., 2012). However, the lack of SS4 is not limiting the formation of starch granules. The formation of starch granules occur independent of SS4 in several mutants. Starch isolated from these mutants analyzed so far did not reveal large inner structural alterations, although the starch morphology reveals considerable differences. Thus, following the starch initiation, the process of starch granule formation is rather similar in these mutants and point to the overlapping functions of the further SSs. Interestingly, the majority of mutants affected in the starch granule number display mostly one starch granule per chloroplast (**Figure 2**). The initiation of starch seems to depend on a chloroplastidial maltodextrin metabolism. At least PTST2 and PHS1 seem to be involved in this process. As this maltodextrin metabolism can be influenced by the ongoing starch turn-over and distinct enzymes of the starch metabolism (e.g., AMY3) the involvement of further proteins/enzymes is highly likely. In yeast cells, starch-like synthesis was recreated by introduction of Arabidopsis SSs without plant enzymes related to synthesis of a primer, thus the intrinsic primers of yeast seems to be sufficient for the formation of starch like polyglucans. Furthermore, the experiments again showed that SS4 can be substituted by other SSs, as starch-like polyglucans were observed not exclusively when SS4 was introduced (Pfister et al., 2016).

## Author Contributions

All authors listed have made a substantial, direct, and intellectual contribution to the work, and approved it for publication.

## Conflict of Interest Statement

The authors declare that the research was conducted in the absence of any commercial or financial relationships that could be construed as a potential conflict of interest.The reviewer NC and handling Editor declared their shared affiliation.

## References

[B1] Ball S. Colleoni C. Cencil I. Raj I. N. Tirtiaux C. (2011). The evolution of glycogen and starch metabolism in eukaryotes gives molecular clues to understanding the establishment of plastid endosymbiosis. *J. Exp. Bot.* 62 1776–1801. 10.1093/jxb/erq411 21220783

[B2] Brust H. Lehmann T. D’Hulst C. Fettke J. (2014). Analysis of the functional interaction of Arabidopsis starch synthase and branching enzyme isoforms reveals that the cooperative action of SSI and BEs results in glucans with polymodal chain lenght distribution similar to amylopectin. *PLoS One* 9:e102364 10.1371/journal.pone.0102364 PMC4094495 25014622

[B3] Brust H. Orzechowski S. Fettke J. Steup M. (2013). Starch synthesizing reactions and paths: *in vitro* and *in vivo* studies. *J. Appl. Glycosci.* 60 3–20. 10.5458/jag.jag.JAG-2012_018

[B4] Chia T. Thorneycroft D. Chapple A. Messerli G. Chen J. Zeeman S. C. (2004). A cytosolic glucosyltransferase is required for conversion of starch in Arabidopsis leaves at night. *Plant J.* 37 853–863. 10.1111/j.1365-313X.2003.02012.x 14996213

[B5] Colleoni C. Linka M. Deschamps P. Handford M. G. Dupree P. Weber A. P. M. (2010). Phylogenetic and biochemical evidence supports the recruitment of an ADP-glucose translocator for the export of photosynthate during plastid endosymbiosis. *Mol. Biol. Evol.* 27 2691–2701. 10.1093/molbev/msq158 20576760

[B6] Comparot-Moss S. Kötting O. Stettler M. Edner C. Graf A. Weise S. E. (2010). A putative phosphatase LSF1 is required for normal starch turnover in Arabidopsis leaves. *Plant Physiol.* 152 685–697. 10.1104/pp.109.148981 20018601 PMC2815883

[B7] Crumpton-Taylor M. Grandison S. Png K. M. Y. Bushby A. J. Smith A. M. (2012). Control of starch granule numbers in Arabidopsis chloroplasts. *Plant Physiol.* 158 905–916. 10.1104/pp.111.186957 22135430 PMC3271777

[B8] Crumpton-Taylor M. Pike M. Lu K. J. Hylton C. M. Feil R. Eicke S. (2013). Starch synthase 4 is essential for coordination of starch granule formation with chloroplast division during Arabidopsis leaf expansion. *New Phytol.* 200 1064–1075. 10.1111/nph.12455 23952675 PMC4283981

[B9] Dauvillée D. Chochois V. Steup M. Haebel S. Eckermann N. Ritte G. (2006). Plastidial phosphorylase is required for normal starch synthesis in *Chlamydomonas reinhardtii*. *Plant J.* 48 274–285. 10.1111/j.1365-313X.2006.02870.x 17018036

[B10] Delvallé D. Dumez S. Wattebled F. Roldan I. Planchot V. Berbezy P. (2005). Soluble starch synthase I: a major determinant for the synthesis of amylpectin in *Arabidopsis thaliana* leaves. *Plant J.* 43 398–412. 10.1111/j.1365-313X.2005.02462.x 16045475

[B11] D’Hulst C. Mérida A. (2012). “Once upon a prime: inception of the understanding of starch initiation in plants,”,” in *Essential Reviews in Experimental Biol Vol. 5 The Synthesis and Breakdown of Starch* ed. Tetlow I. J. (London: The Society for Experimental Biology) 55–76.

[B12] Fettke J. Chia T. Eckermann N. Smith A. M. Steup M. (2006). A transglucosidase necessary for starch degradation and maltose metabolism in leaves at night acts on cytosolic heteroglycans (SHG). *Plant J.* 46 668–684. 10.1111/j.1365-313X.2006.02732.x 16640603

[B13] Fettke J. Fernie A. R. Steup M. (2012a). “Transitory starch and its degradation in higher plant cells,” in *Essential Reviews in Experimental Biology: Starch: Origins, Structure and Metabolism* Vol. 5 ed. Tetlow I. J. (London: SEB) 311–374.

[B14] Fettke J. Leifels L. Brust H. Herbst K. Steup M. (2012b). Two carbon fluxes to reserve starch in potato (*Solanum tuberosum* L.) tuber cells are closely interconnected but differently modulated by temperature. *J. Exp. Bot.* 63 3011–3029. 10.1093/jxb/ers014 22378944 PMC3350916

[B15] Gamez-Arjona F. M. Li J. Raynaud S. Baroja-Fernanddez E. Munoz F. J. Ovecka M. (2011). Enhancing the expression of starch synthase class IV results in increased levels of both transitory and longterm storage starch. *Plant Biotechnol. J.* 9 1049–1060. 10.1111/j.1467-7652.2011.00626.x 21645200

[B16] Gamez-Arjona F. M. Raynaud S. Ragel P. Merida A. (2014). Starch synthase 4 is located in the thylakoid membrane and interacts with plastoglobule-associated proteins in Arabidopsis. *Plant J.* 80 305–316. 10.1111/tpj.12633 25088399

[B17] Hejazi M. Fettke J. Steup M. (2012). “Starch phosphorylation and dephosphorylation: the consecutive action of starch-related dikinases and phosphatases,” in *Essential Reviews in Experimental Biology: Starch: Origins, Structure and Metabolism* Vol. 5 ed. Tetlow I. J. (London: SEB) 279–308.

[B18] Hwang S. K. Koper K. Satoh H. Okita T. W. (2016). Rice endosperm starch phosphorylase (Pho1) assembles with disproportionating enzyme (Dpe1) to form a protein complex that enhances synthesis of malto-oligosaccharides. *J. Biol. Chem.* 291 19994–20007. 10.1074/jbc.M116.735449 27502283 PMC5025686

[B19] Kötting O. Santelia D. Edner C. Eicke S. Marthaler T. Gentry M. S. (2009). STARCH-EXCESS4 is a laforin-like phosphoglucan phosphatase required for starch degradation in *Arabidopsis thaliana*. *Plant Cell* 21 334–346. 10.1105/tpc.108.064360 19141707 PMC2648081

[B20] Leterrier M. Holappa L. D. Broglie K. E. Beckler D. M. (2008). Cloning, characterisation and comparative analysis of a starch synthase IV gene in wheat: functional and evolutionary implications. *BMC Plant Biol.* 8 98–119. 10.1186/1471-2229-8-98 18826586 PMC2576272

[B21] Lu K. J. Pfister B. Jenny C. Eicke S. Zeeman S. C. (2018). Distinct functions of starch synthase 4 domains in starch granule formation. *Plant Physiol.* 176 566–581. 10.1104/pp.17.01008 29133376 PMC5761780

[B22] Lu Y. Sharkey T. D. (2004). The role of amylomaltase in maltose metabolism in the cytosol of photosynthetic cells. *Planta* 218 466–473. 10.1007/s00425-003-1127-z 14593480

[B23] Mahlow S. Hejazi M. Kuhnert F. Garz A. Brust H. Baumann O. (2014). Phosphorylation of transitory starch by α-glucan, water dikinase during starch turnover affects the surface properties and morphology of starch granules. *New Phytol.* 203 495–507. 10.1111/nph.12801 24697163

[B24] Mahlow S. Orzechowski S. Fettke J. (2016). Starch phosphorylation: insights and perspectives. *Cell. Mol. Life Sci.* 73 2753–2764. 10.1007/s00018-016-2248-4 27147464 PMC11108486

[B25] Malinova I. Alseekh S. Feil R. Fernie A. R. Baumann O. Schöttler M. A. (2017). Starch synthase 4 and plastidal phosphorylase differentially affect starch granule number and morphology. *Plant Physiol.* 174 73–85. 10.1104/pp.16.01859 28275148 PMC5411139

[B26] Malinova I. Fettke J. (2017). Reduced starch granule number per chloroplast in the *dpe2/phs1* mutant is dependent on initiation of starch degradation. *PLoS One* 12:e0187985 10.1371/journal.pone.0187985 PMC5695794 29155859

[B27] Malinova I. Mahlow S. Alseekh S. Orawetz T. Fernie A. R. Baumann O. (2014). Double knock-out mutants of Arabidopsis thaliana grown under normal conditions reveal that the plastidial phosphorylase isozyme (PHS1) participates in transitory starch metabolism. *Plant Physiol.* 164 907–921. 10.1104/pp.113.227843 24302650 PMC3912115

[B28] Nakamura Y. (2015). “Initiation process of starch biosynthesis,” in *Starch: Metabolism and Structure* ed. Nakamura Y. (Tokyo: Springer) 315–332.

[B29] Nakamura Y. Ono M. Sawada T. Crofts N. Fujita N. Steup M. (2017). Characterization of the functional interactions of plastidial starch phosphorylase and starch branching enzymes from rice endosperm during reserve starch biosynthesis. *Plant Sci.* 264 83–95. 10.1016/j.plantsci.2017.09.002 28969805

[B30] Niittylä T. Messerli G. Trevisan M. Chen J. Smith A. M. Zeeman S. C. (2004). A previously unknown maltose transporter essential for starch degradation in leaves. *Science* 303 87–89. 10.1126/science.1091811 14704427

[B31] Orawetz T. Malinova I. Orzechowski S. Fettke J. (2016). Reduction of the plastidial phosphorylase in potato (*Solanum tuberosum* L.) reveals impact on storage starch structure during growth at low temperature. *Plant Physiol. Biochem.* 100 141–149. 10.1016/j.plaphy.2016.01.013 26828405

[B32] Pfister B. Sanchez-Ferrer A. Diaz A. Lu K. J. Otto C. Holler M. (2016). Recreating the synthesis of starch granules in yeast. *eLife* 5:e15552 10.7554/eLife.15552 PMC5119888 27871361

[B33] Qasim H. M. (2018). *Protein Interactions During Starch Granule Initiation in Arabidopsis thaliana.* Ph.D. thesis report. Potsdam: University of Potsdam.

[B34] Ragel P. Streb S. Feil R. Sahrawy M. Annunziata M. G. Lunn J. E. (2013). Loss of starch granule initiation has a deleterious effect on the growth of Arabidopsis plants due to an accumulation of ADP-glucose. *Plant Physiol.* 163 75–85. 10.1104/pp.113.223420 23872660 PMC3762666

[B35] Raynaud S. Ragel P. Rojas T. Merida A. (2016). The N-terminal part of *Arabidopsis thaliana* starch synthase 4 determines the localization and activity of the enzyme. *J. Biol. Chem.* 291 10759–10771. 10.1074/jbc.M115.698332 26969163 PMC4865922

[B36] Ritte G. Lloyd J. R. Eckermann N. Rottmann A. Kossmann J. Steup M. (2002). The starch-related R1 protein is an alpha-glucan water dikinase. *Proc. Natl. Acad. Sci. U.S.A.* 99 7166–7171. 10.1073/pnas.062053099 12011472 PMC124546

[B37] Roldán I. Lucas M. M. Delvalle D. Planchot V. Jimenez S. Perez R. (2007). The phenotype of soluble starch synthase IV defective mutants of *Arabidopsis thaliana* suggests a novel function of elongation enzymes in the control of starch granule formation. *Plant J.* 492–504. 10.1111/j.1365-313X.2006.02968.x 17217470

[B38] Satoh H. Shibahara K. Tokunaga T. Nishi A. Tasaki M. Hwang S. K. (2008). Mutation of the plastidial alpha-glucan phosphorylase gene in rice affects the synthesis and structure of starch in the endosperm. *Plant Cell* 20 1833–1849. 10.1105/tpc.107.054007 18621947 PMC2518224

[B39] Seung D. Boudet J. Monroe J. Schreier T. B. David L. C. Abt M. (2017). Homologs of PROTEIN TARGETING TO STARCH control starch granule initiation in Arabidopsis leaves. *Plant Cell* 29 1657–1677. 10.1105/tpc.17.00222 28684429 PMC5559754

[B40] Seung D. Lu K. J. Stettler M. Streb S. Zeeman S. C. (2016). Degradation of glucan primers in the absence of starch synthase 4 disrupts starch granule initiation in *Arabidopsis*. *J. Biol. Chem.* 291 20718–20728. 10.1074/jbc.M116.730648 27458017 PMC5034061

[B41] Seung D. Soyk S. Coiro M. Maier B. A. Eicke S. Zeeman S. C. (2015). PROTEIN TARGETING TO STARCH is required for localizing GRANULE-BOUND STARCH SYNTHASE to starch granules and for normal amylose synthesis in Arabidopsis. *PLoS Biol.* 13:e1002080 10.1371/journal.pbio.1002080 PMC4339375 25710501

[B42] Szydlowski N. Ragel P. Hennen-Bierwagen T. A. Planchot V. R. Myers A. M. Merida A. (2011). Integrated functions among multiple starch synthases determine both amylopectin chain length and branch linkage location in *Arabidopsis* leaf starch. *J. Exp. Bot.* 62 4547–4559. 10.1093/jxb/err172 21624979

[B43] Szydlowski N. Ragel P. Raynaud S. Lucas M. M. Roldan I. Montero M. (2009). Starch granule initiation in *Arabidopsis* requires the presence of either class IV or class III starch synthases. *Plant Cell* 21 2443–2457. 10.1105/tpc.109.066522 19666739 PMC2751949

[B44] Tetlow I. J. Wait R. Lu Z. Akkasaeng R. Bowsher C. G. Esposito S. (2004). Protein phosphorylation in amyloplasts regulates starch branching enzyme activity and protein-protein interactions. *Plant Cell* 16 694–708. 10.1105/tpc.017400 14973170 PMC385281

[B45] Ugalde J. E. Parodi A. J. Ugalde R. A. (2003). De novo synthesis of bacterial glycogen: *Agrobacterium tumefaciens* glycogen synthase is involved in glucan initiation and elongation. *Proc. Natl. Acad. Sci. U.S.A* 100 10659–10663. 10.1073/pnas.1534787100 12960388 PMC196860

[B46] Zeeman S. C. Thorneycroft D. Schupp N. Chapple A. Weck M. Dunstan H. (2004). Plastidial α-glucan phosphorylase is not required for starch degradation in Arabidopsis leaves but has a role in the tolerance of abiotic stress. *Plant Physiol.* 135 849–858. 10.1104/pp.103.032631 15173560 PMC514120

[B47] Zhang X. Myers A. M. James M. G. (2005). Mutations affecting starch synthase III in Arabidopsis alter leaf starch structure and increase the rate of starch synthesis. *Plant Physiol.* 138 663–674. 10.1104/pp.105.060319 15908598 PMC1150387

[B48] Zhang X. Szydlowski N. Delvalle D. D’Hulst C. James M. Myers A. (2008). Overlapping functions of the starch synthases SSII and SSIII in amylopectin biosynthesis in *Arabidopsis*. *BMC Plant Biol.* 8:96 10.1186/1471-2229-8-96 PMC2566982 18811962

